# Highly efficient free-breathing 3D whole-heart imaging in 3-min: single center study in adults with congenital heart disease

**DOI:** 10.1016/j.jocmr.2023.100008

**Published:** 2023-12-22

**Authors:** Anastasia Fotaki, Kuberan Pushparajah, Christopher Rush, Camila Munoz, Carlos Velasco, Radhouene Neji, Karl P. Kunze, René M. Botnar, Claudia Prieto

**Affiliations:** a Department of Biomedical Engineering, School of Biomedical Engineering and Imaging Sciences, King’s College London, St Thomas’ Hospital, SE1 7EH London, United Kingdom; b Guy’s and St Thomas’ NHS Foundation Trust, London, United Kingdom; c MR Research Collaborations, Siemens Healthcare Limited, Frimley, United Kingdom; d Escuela de Ingeniería, Pontificia Universidad Católica de Chile, Santiago, Chile; e Institute for Biological and Medical Engineering, Pontificia Universidad Católica de Chile, Santiago, Chile; f Millennium Institute for Intelligent Healthcare Engineering, Santiago, Chile; g Institute for Advanced Study, Technical University of Munich, Lichtenbergstrasse 2 a, D-85748 Garching, Germany

**Keywords:** INAV, Low-rank, 3D whole-heart, Congenital heart disease

## Abstract

**Background:**

Three dimensional, whole-heart (3DWH) MRI is an established non-invasive imaging modality in patients with congenital heart disease (CHD) for the diagnosis of cardiovascular morphology and for clinical decision making. Current techniques utilise diaphragmatic navigation (dNAV) for respiratory motion correction and gating and are frequently limited by long acquisition times. This study proposes and evaluates the diagnostic performance of a respiratory gating-free framework, which considers respiratory image-based navigation (iNAV), and highly accelerated variable density Cartesian sampling in concert with non-rigid motion correction and low-rank patch-based denoising (iNAV-3DWH-PROST). The method is compared to the clinical dNAV-3DWH sequence in adult patients with CHD.

**Methods:**

In this prospective single center study, adult patients with CHD who underwent the clinical dNAV-3DWH MRI were also scanned with the iNAV-3DWH-PROST. Diagnostic confidence (4-point Likert scale) and diagnostic accuracy for common cardiovascular lesions was assessed by three readers. Scan times and diagnostic confidence were compared using the Wilcoxon-signed rank test. Co-axial vascular dimensions at three anatomic landmarks were measured, and agreement between the research and the corresponding clinical sequence was assessed with Bland-Altman analysis.

**Results:**

The study included 60 participants (mean age ± [SD]: 33 ± 14 years; 36 men). The mean acquisition time of iNAV-3DWH-PROST was significantly lower compared with the conventional clinical sequence (3.1 ± 0.9 min vs 13.9 ± 3.9 min, p < 0.0001). Diagnostic confidence was higher for the iNAV-3DWH-PROST sequence compared with the clinical sequence (3.9 ± 0.2 vs 3.4 ± 0.8, p < 0.001), however there was no significant difference in diagnostic accuracy. Narrow limits of agreement and mean bias less than 0.08 cm were found between the research and the clinical vascular measurements.

**Conclusions:**

The iNAV-3DWH-PROST framework provides efficient, high quality and robust 3D whole-heart imaging in significantly shorter scan time compared to the standard clinical sequence.

## 1 Background

Congenital heart disease (CHD) refers to structural heart disease and is characterized by extremely wide anatomical variations and range of complexity. Over the last decades, surgical, interventional and supportive care in patients with CHD has made significant strides leading to longer life expectancy and improved quality of life [1]. Accurate demonstration of the cardiovascular anatomy is essential for the understanding of the pathology and clinical decision making. Three dimensional, whole-heart (3DWH) Magnetic Resonance Angiography (MRA) is an established non-invasive imaging modality in this patient population [2]. It is utilised for the diagnosis of cardiovascular morphology and pre-procedural planning and follow-up. Despite its significant clinical merit, conventional clinical protocols have still not adopted recent advances in the field of 3DWH MRA and can suffer from long and unpredictable scan times and residual motion artefacts affecting image quality [2], [3].

The current clinical acquisition and reconstruction protocols include ∼2-fold uniform Cartesian undersampling with parallel imaging reconstruction [2], [4], diaphragmatic gating for respiratory motion correction [3] and T2 preparation pulses, such as the Malcolm-Levitt phase cycling 2 (MLEV2) [5] pulse to improve contrast between blood and myocardium. The narrow diaphragmatic gating window limits acceleration of the acquisition time because only data acquired at the quiescent part of the respiratory cycle (usually end-expiration) are utilised for image reconstruction. Furthermore, the simplified respiratory correction model, which uses a fixed correction factor of 0.6, does not fully account for the complex relationship between respiratory-induced diaphragmatic motion and the motion of the heart, often introducing unresolved respiratory motion artefacts, particularly in subjects with irregular breathing patterns [6], [7], [8]. These, along with the uncorrected non-rigid respiratory-induced cardiac motion can further impede image quality [9]. Additionally, the commonly used MLEV2 T2 preparation pulse poses further challenges, as it is susceptible to flow-related and off-resonance artefacts [10], that may be frequent in this population, as a result of the underlying anatomy (turbulent flow in areas of stenosis or regurgitation) and the proximity of structures of interest to the lung interface [2], [9], [10].

Several approaches utilizing 1D self-navigation and 2D/3D image-based navigators (iNAVs) have been proposed to correct for respiratory motion and utilize almost all the acquired data for reconstruction, thereby achieving ∼100% respiratory scan efficiency, and thus shorter and predictable scan time [11], [12], [13], [14]. Despite the improved respiratory scan efficiency, the fully sampled 3DWH acquisition is still lengthy because the data acquisition is typically electrocardiogram (ECG) triggered with short acquisition window ∼100–120 ms to minimize the effect of cardiac motion. Therefore, undersampled trajectories in concert with advanced reconstruction techniques have been proposed to produce images of acceptable quality from undersampled data. Research efforts have explored parallel imaging and the inherent redundancies of the MR images or the k-space in the spatial or motion domain to recover the information that was not sampled. Studies utilising these techniques in 3DWH imaging for thoracic vasculature resulted in a mean acquisition time of 6–7 min for a resolution of 1.5–1.7 mm^3^ [15], [16], [17], however some of these are limited by long reconstruction times and the requirement for increased computational demand [16], [17].

A previously introduced non-rigid respiratory motion corrected 3DWH framework has demonstrated promising results in coronary MRA [18]. This approach employs pseudo-random, highly-accelerated variable-density acquisition (VD-CASPR) [19], with four-fold acceleration, iNAV based motion correction and Patch-based RecOnSTruction (PROST), which exploits spatial correlations in the local and non-local scale from VD-CASPR undersampled data. Additionally, a recent study integrating part of this framework, including iNAV in concert with three-fold acceleration and conventional iterative sensitivity encoding (it-SENSE) reconstruction, has demonstrated attenuation of artefacts in aortic imaging in a fraction of the clinical acquisition time [9]. Nevertheless, the diagnostic performance of a sequence encompassing all of the above features has not been validated for the anatomical evaluation of the heart and the thoracic vasculature in adults with CHD.

This work proposes to amalgamate the aforementioned advances in a framework for contrast-agent free, 3DWH imaging in CHD, which incorporates 4-fold accelerated acquisition with VD-CASPR undersampling in concert with iNAV-based non-rigid motion correction and low-rank patch-based based denoising (PROST) for efficient, high quality 3D whole-heart imaging. We hypothesized that this framework can perform at least as well as the clinical sequence, albeit in a fraction of the acquisition time.

## 2 Methods

### 2.1 Study design

This was a prospective single center study. This study was performed in accordance with the Declaration of Helsinki and approved by the National Research Ethics Service (REC 15/NS/0030). Written informed consent was obtained from each participant according to institutional guidelines.

### 2.2 Study participants

Sixty consecutive patients with CHD referred for a clinical cardiac MRI examination were recruited for this study between January 2022 and September 2022. The diagnosis of CHD was described in the clinical referral form. Patients were eligible to participate if they were > 18 years of age and consented to the study. Specific exclusion criteria were contraindications for cardiac MRI (e.g., pacemaker, cochlear implants, cerebral aneurysm clip, implanted electronic device, or claustrophobia) and inability to lie flat. Details about the patient cohort and corresponding diagnoses are presented in Table 1.

**Table 1:** Participant characteristics.

No. of participants	60
Age (y)^†^	33 ± 14 years
Sex	
Men	36/60 (60%)
Women	24 /60(40%)
Weight (kg)^†^	77 ± 18
Height (m)^†^	1.77 ± 0.12
BMI^†^(kg/m^2^)	26 ± 5.8
Heart Rate* (beats per minute)	69 ± 12 (40,96)
Indication	
Aortic disease (including aortic valve and thoracic aorta disease, e.g. bicuspid aortic valve, coarctation, aneurysm)	14/60 (23%)
Tetralogy of Fallot	8/60 (13%)
Atrioventricular septal defect	5/60 ( 8%)
Transposition of the great arteries	4/60 (7%)
Hypoplastic left heart syndrome	4/60 (7%)
Ventricular septal defect	3/60 (5%)
Pulmonary stenosis	3/60 (5%)
Anomalous pulmonary venous drainage	3/60 (5%)
Pulmonary atresia	2/60 (3%)
Transposition of the great arteries	2/60 (3%)
Secundum atrial septal defect	2/60 (3%)
Congenitally corrected transposition of the great arteries	2/60 (3%)
Tricuspid valve abnormalities	2/60 (3%)
Ebstein’s anomaly	1/60 (2%)
Shone’s complex	1/60 (2%)
Mitral valve abnormalities	1/60 (2%)
Double-outlet right ventricle	1/60 (2%)
Tricuspid and pulmonary atresia	1/60 (2%)
Scimitar syndrome	1/60 (2%)

† Numbers are means ± standard deviations.

* Numbers are me ± standard deviations, with ranges in parentheses.

Note: — Except where indicated, data are numbers of participants, with percentages in parentheses. BMI: Body Mass Index.

### 2.3 Cardiac MRI protocol

All acquisitions reported in this study were performed on a 1.5T system (MAGNETOM Aera, Siemens Healthineers, Erlangen, Germany) using an 18-channel chest-coil and a 32-channel spine coil. The recruited participants underwent 3D whole-heart imaging with the clinically requested dNAV-3DWH acquisition and the research iNAV-3DWH-PROST sequence. The iNAV-3DWH-PROST was acquired at the end of the imaging session, after the clinical protocol. No contrast-agent was administered in any of the exams.

### 2.4 iNAV-3DWH-PROST framework

Data acquisition with the proposed sequence (iNAV-3DWH-PROST) consisted of an ECG-triggered 3D balanced steady-state free precession (bSSFP) research sequence (Fig. 1.1). The T2 preparation module consisted of a 90° rectangular excitation pulse, followed by a train of four equally spaced refocusing modules (MLEV4) [5], [20] and a 90°tip-up rectangular pulse. 2D iNAVs were acquired at each heartbeat by spatially encoding the start-up echoes of the bSSFP acquisition to enable 100% respiratory efficiency (without data rejection) and predictable scan time. To further reduce scan time, 3D data were acquired with a 4-fold undersampled variable-density golden-step Cartesian trajectory with spiral profile order sampling [21]. This introduced incoherently distributed, noise-resembling perturbations in the spatial dimension, which are suitable for low-rank based denoising. The iNAVs were used to estimate the respiratory signal, so that by tracking a rectangular template located around the heart, translational superior-inferior (SI) and right-left (RL) respiratory motion can be obtained [12]. The translational respiratory motion corrected data were used in a research reconstruction implemented inline on the scanner to produce non-rigid motion-corrected datasets as previously described [22]. Briefly, the SI motion was used to group the acquired data into equally populated respiratory bins, and binned 3D data was corrected for translational SI and RL respiratory motion to the center of the corresponding bin (Fig. 1.2). Respiratory-resolved bin images were then reconstructed with it-SENSE at half of the full imaging resolution, histogram-equalized, and subsequently used to estimate the non-rigid bin-to-bin respiratory motion fields (Fig. 1.2). The non-rigid deformation fields were then interpolated back to full image resolution and were incorporated into a generalized matrix formulation for motion-corrected MR reconstruction [22] (Fig. 1.3). To further minimize artifacts arising from the undersampled acquisition, a low-rank patch-based (PROST) denoising [23] was performed after motion-corrected reconstruction (Fig. 1.3). The described motion-compensated reconstruction including respiratory binning and non-rigid motion correction was performed in-line on the scanner. Low-rank patch-based denoising was performed offline in MATLAB (The MathWorks, Natick, Massachusetts, USA) on a workstation with a 16-core Dual Intel Xeon Processor (23 GHz, 256 GB RAM).

**Fig. 1:**
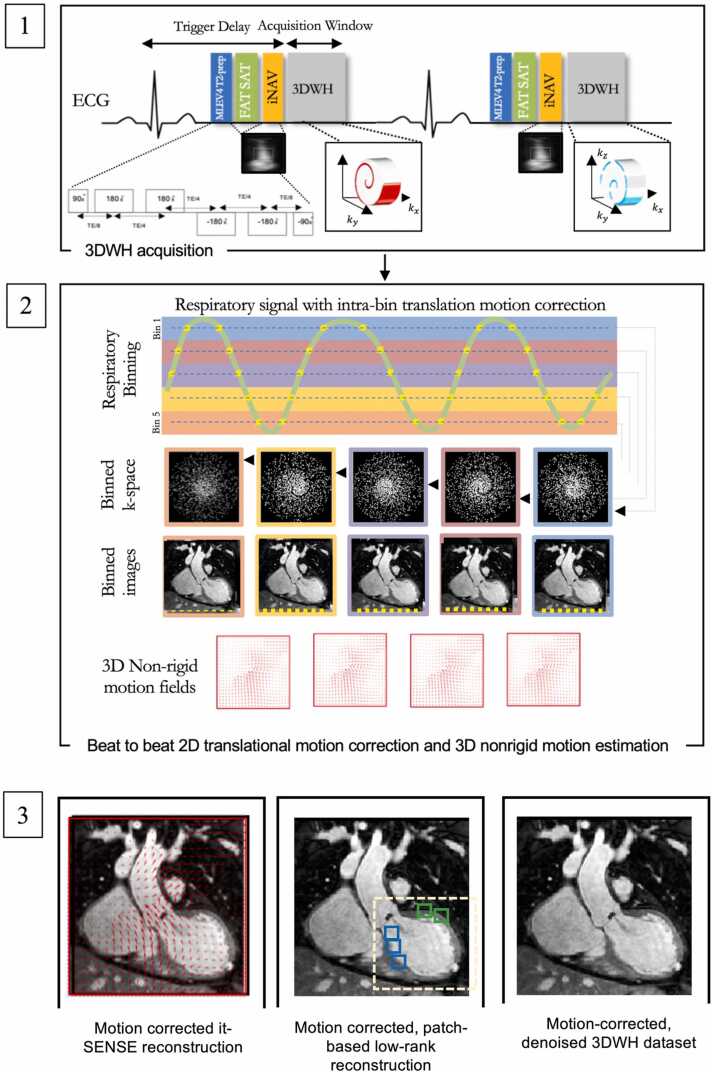
**Schematic overview of investigated free-breathing non-rigid motion-corrected iNAV-3DWH-PROST framework**. Schematic overview of investigated free-breathing non-rigid motion-corrected iNAV-3DWH-PROST framework. MLEV4 T2 preparation pulse precedes the acquisition in each heartbeat (1.1). Frequency‐selective pre‐saturation is used to suppress the signal from epicardial fat (1.1). Data acquisition is performed using a 3D Cartesian trajectory with spiral-like profile order (1.1). A low‐resolution two-dimensional (2D) iNAV is acquired in each heartbeat by spatially encoding the ramp‐up pulses of the bSSFP sequences (1.1). The iNAVs are used to estimate superior-inferior and right-left rigid motion by tracking a template around the heart, providing motion estimates in a beat-to-beat basis. (1.2) Superior-inferior motion is used to sort the 3D data into 5 equally populated bins and 3D MRIs reconstructed at each respiratory position are used to estimate non-rigid motion between bins. 2D translational beat-to-beat and 3D non-rigid bin-to-bin motion is then integrated into an in-line motion-compensated iterative sensitivity encoding (SENSE) reconstruction to produce the motion-corrected images (1.3). Low-rank patch-based (PROST) denoising is performed after motion-corrected reconstruction to generate the final dataset (Figure 1.3). Abbreviation:iNAV: image-based navigator, kx: readout, ky: phase encoding, MLEV: Malcolm Levitt cycling, SENSE: sensitivity encoding, 3DWH: 3D whole-heart.

### 2.5 Experiments

The clinical protocol included a standard dNAV-3DWH sequence, acquired with the following sequence parameters: T2-prepared bSSFP readout, sagittal orientation, field of view (FOV) = 400 × 256 × 134–202 mm^3^, spatial resolution = 1.5 mm^3^ isotropic, T2Prep duration = 40 ms, GRAPPA parallel imaging 2 × undersampled, flip angle = 90º, repetition time (TR)/echo time (TE) = 3.3/1.6 ms, bandwidth = 557 Hz/px. These acquisitions were performed under free breathing, with diaphragmatic navigator gating for respiratory motion compensation, with a gating window of ± 3.5 mm in end-expiration and a slice tracking factor of 0.6 (Supplementary Table 1).

After the clinical scan, data were acquired with the proposed iNAV-3DWH-PROST method, with the following parameters: bSSFP readout, coronal orientation, FOV = 400 × 300 × 104–208 mm^3^, spatial resolution = 1.5 mm^3^ isotropic, 4-fold undersampling, flip angle = 90º, TR/TE = 3.4/1.7 ms bandwidth = 919 Hz/px. For both the clinical and the proposed acquisitions, a subject-specific trigger delay and acquisition window (120–150 ms) were set to coincide with ventricular mid-diastole to minimize the effect of cardiac motion (Supplementary Table 1). These acquisition parameters were estimated by visually inspecting the motion of the coronary artery in a free-breathing 4-chamber cine image, acquired with 3 signal averages and 50 phases. The middiastolic rest period was defined from cessation of motion of the right coronary artery (corresponding to the end of early ventricular filling) to the beginning of atrial systole. To minimise breathing related artefacts the fold-over direction was chosen in the LR instead of the anterior-posterior (AP) direction. Scan time for all sequences, in-line reconstruction time and off-line denoising time for the iNAV-3DWH-PROST was recorded.

### 2.6 Image quality analysis

#### 2.6.1 Visual analysis

Image processing and reformatting was performed with commercially available analysis software (Horos, version 1.1.7). 120 datasets were anonymized, de-identified and randomized for evaluation. Blinded to participant characteristics and the other readers’ evaluations, three cardiologists who specialize in cardiac MRI for ACHD [1 (KP), 2 (CR), 3 (AF)]; all European Association of Cardiovascular Imaging (EACVI) Level 3 accreditation with 15, 8 and 4 years of experience, respectively) independently reviewed 20 separate datasets from each sequence (20 ×3 readers x 2 sequences = 120 total datasets). Before visual evaluation, the readers were given training data sets with poor to excellent image quality to calibrate their scores together. Each reviewer scored the image quality of all intrapericardial structures. Image quality assessment was based on a 5-point scoring system and was divided on sharpness of vessel or cardiac wall borders (1:non diagnostic- 5:excellent) and robustness to artifact (1: severe artifact-5: minimal artifact) [24]. Additionally, each clinician assessed the datasets for the presence of the following abnormalities: 1) main pulmonary artery stenosis, 2) right pulmonary artery stenosis, 3) left pulmonary artery stenosis, 4) coronary artery abnormalities (course or stenosis), 5) coarctation of the aorta 6) aortic dilatation/aneurysm, 7) abnormal aortic arch anatomy (including presence of large aorta-pulmonary collaterals) and 8) anomalous pulmonary venous drainage (total or partial). Each abnormality was scored on a 5-point Likert scale (1 = definitely not present, 2 = probably not present, 3 = unclear, 4 = probably present, 5 = definitely present), allowing evaluation of diagnostic sensitivity and specificity. For diagnostic sensitivity and specificity, scores of 1 and 2 were coded as absent, and 4 and 5 were coded as present. A score of 3 was coded as a misdiagnosis [25]. Subsequently, the reviewers scored their diagnostic confidence to perform sequential segmental analysis with each dataset using a 4-point Likert scale (1:low confidence, 2:moderate but additional imaging required, 3:high (diagnostic), 4:definite). After grading diagnostic confidence, the MRI findings could be adjudicated with locally available echocardiographic, catheterisation, Computed Tomography, and operative data. When these data were not available, independent review by two experts (consultant cardiologists specialized in ACHD and in MRI in ACHD, each with 15 years of experience), blinded to participant information was obtained. The data scoring criteria are detailed in Supplementary Table 2.

### 2.7 Quantitative image quality analysis

The contrast ratio between blood and myocardium was computed in the respective intrapericardial structures. For this, circular regions of interest (ROIs) were manually placed by reader 3 in the left ventricular myocardium at mid-ventricular level (area of 25 mm^2^) and the intrapericardial structures (area of 20–40 mm^2^ diameter) at the same anatomical locations using Osirix (version 9.0; OsiriX Foundation, Geneva, Switzerland). Blood-to-myocardium contrast ratio (CR) was computed at each location as (µ_"ROI,lumen" - µ_"ROI,myocardium")⁄( µ_"ROI,myocardium") where µROI corresponded to the mean value in each ROI.

### 2.8 Measurement reproducibility analysis

Co-axial measurements (maximum diameter) were performed using multiplanar reformats by reviewer 2 and 3 [26]**.** Vascular dimensions were measured at three landmarks predefined by literature guidelines^17^, namely the aortic root, mid left and mid right pulmonary artery by two of the readers. The readers were blinded to the underlying diagnosis and patient demographics. Reviewers 2 and 3 analyzed 20 separate, paired datasets. Measurements were used for comparison between the research and clinical sequence. Reviewer 3 also analyzed the sample of the 20 research datasets, that was analyzed by reviewer 2, to assess for inter-observer reliability. Reviewer 3 repeated the measurements in 20 iNAV-3DWH-PROST datasets after a 2-month interval to assess intra-observer reliability.

### 2.9 Qualitative analysis of the research framework pre- and post-PROST denoising

The research datasets were analysed pre- (iNAV-3DWH) and post-PROST denoising (iNAV-3DWH-PROST) in terms of sharpness of vessel or cardiac wall borders (1: non diagnostic- 5:excellent) and robustness to artifact (1:severe artifact- 5:minimal artifact)along with diagnostic performance, to investigate the benefit of incorporating PROST in the framework.

### 2.10 Statistical analysis

Continuous variables were presented as mean ± standard deviation. In view of the sample size (N = 60), normal approximation to the means was adequate due to the central limit theorem. The Student-t test was used to compare differences in continuous variables between two groups. Subjective scores were compared with a paired Wilcoxon-signed-rank test to assess statistical differences. For the comparison of the three datasets, a one-way analysis of variance was used. Two-tailed values of P < 0.05 were considered statistically significant differences. The intra-class correlation coefficient was used to assess intra- and inter-observer variability; Bland Altman analysis was used to assess inter-observer variability and agreement between the research and the clinical methods. Statistical analysis was performed using Graphpad Prism (Version 9.1.0, Graph-Pad Software, San Diego, California, USA).

## 3 Results

### 3.1 Participant characteristics

Imaging was successfully completed in all 60 consecutive participants (mean age, 33 ± 14 years; 36 men). Participant baseline characteristics are listed in Table 1.

### 3.2 3D Whole-heart magnetic resonance imaging analysis

The mean acquisition time of iNAV-3DWH-PROST was significantly shorter compared with the conventional clinical sequence (3.1 ± 0.9 min vs 13.9 ± 3.9 min, p < 0.001). In-line reconstruction time for the iNAV-3DWH-PROST was 2.6 ± 0.7 min and off-line denoising was 0.9 ± 0.2 min. Total reconstruction time was 2.9 ± 0.4 min

**Fig. 2:**
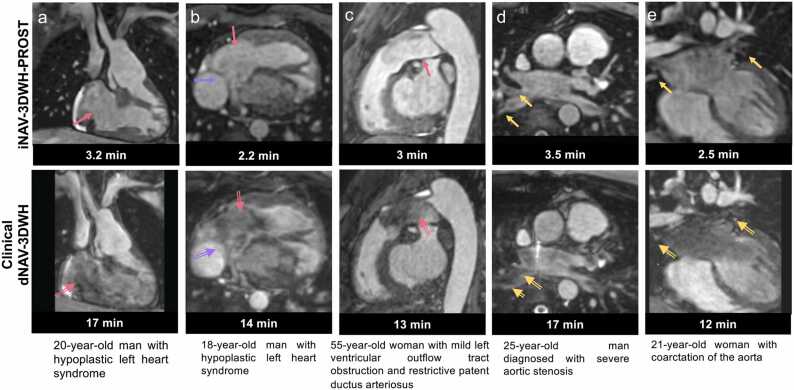
Visual comparison of images from the research and clinical datasets from selected patients. Comparison of research iNAV-3DWH-PROST and clinical dNAV-3DWH has demonstrated significant scan time reduction and attenuation of artefacts: **a.** Multiplanar reformatted images in 20-year-old man diagnosed with hypoplastic left heart syndrome post Fontan palliation (lateral tunnel with fenestration). Artifacts are minimised with the proposed framework (pink arrow). Slow flow has caused signal voids in the right atrium and right ventricle (pink blank arrow) in the clinical sequence, b: Multiplanar reformatted images in 18-year-old man with hypoplastic left heart syndrome post-total cavo-pulmonary connection completion with a fenestrated lateral tunnel Fontan pathway. Signal voids are observed in the right atrium due to stagnant flow (pink blank arrow) in the clinical dataset and artefact from respiratory motion, impeded the visualisation of the fenestration (purple blank arrow). The artefacts are attenuated with the iNAV-3DWH-PROST (pink and purple arrows respectively), **c**. Multiplanar reformatted images in 55-year-old man with restrictive patent ductus arteriosus and mild left ventricular outflow tract obstruction. Signal voids due to artefacts from turbulent flow are present in clinical dataset (pink blank arrows) and reduced with the proposed approach (pink arrow), **d:** Multiplanar reformatted images in 25-year-old man with severe aortic stenosis. Substantial off-resonance artefacts in the pulmonary veins are observed with the clinical dNAV-3DWH (yellow blank arrows), which impede the sequential segmental anatomical description. Pulmonary venous return can be established in the research iNAV-3DWH-PROST dataset (yellow arrows), **e:** Multiplanar reformatted images in 21-year-old woman with coarctation of the aorta. Off-resonance artefacts in the pulmonary veins in the clinical sequence (yellow blank arrows) are attenuated with the iNAV-3DWH-PROST (yellow arrows).

**Fig. 3:**
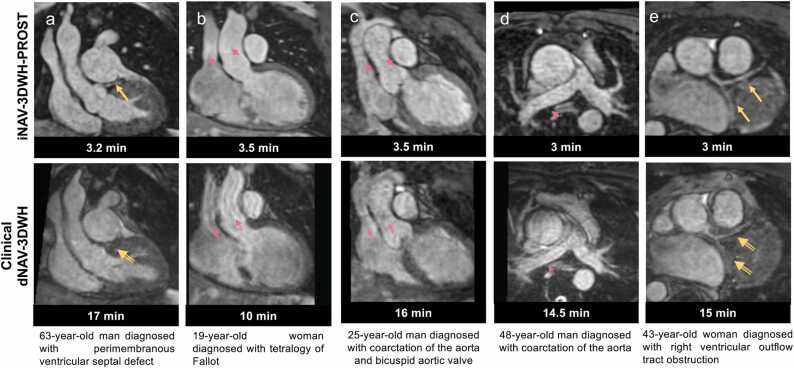
Visual comparison of images from the research and clinical datasets from selected patients. Comparison of research iNAV-3DWH-PROST and clinical dNAV-3DWH has demonstrated significant scan time reduction and attenuation of artefacts: **a.** Multiplanar reformatted images in 63-year-old man diagnosed with perimembranous ventricular septal defect. The left anterior descending coronary artery is well demarcated with the iNAV-3DWH-PROST (yellow arrow). Residual non-rigid motion artefacts hinders diagnostic certainty in the clinical sequence (yellow blank arrow), **b:** Multiplanar reformatted images in 19-year-old woman diagnosed with tetralogy of Fallot. Residual respiratory motion causes substantial blurring in the ascending aorta and superior vena cava (pink blank arrows) in the clinical dataset. The iNAV-3DWH resolves the respiratory motion in the respective structures (pink arrows), **c:** Multiplanar reformatted images in 25-year-old man with coarctation of the aorta and bicuspid aortic valve. Substantial artefacts from respiratory motion in the clinical datasets (pink blank arrows) are attenuated with the research sequence (pink arrows), **d:** Multiplanar reformatted images in 48-year-old man with coarctation of the aorta. INAV-3DWH-PROST resolves the respiratory motion and flow-artefacts, enabling accurate measurements of the ascending aorta and branch pulmonary arteries. Significant artefacts from respiratory motion in the clinical sequence hinders reliable dimensioning (pink blank arrows), **e:** Multiplanar reformatted images in 43-year-old woman with right ventricular outflow tract obstruction. Blurring in the delineation of the coronary arteries, due to residual non-rigid motion in the clinical dataset (yellow blank arrows), is attenuated with the iNAV-3DWH-PROST (yellow arrows).

A spectrum of complex morphologic features and vascular anatomy was clearly delineated with iNAV-3DWH-PROST as shown in Fig. 2, Fig. 3 and Supplementary Fig. 1–2, in comparison to the clinical sequence. In participants with low flow/flow stagnation (Fontan pathway) (Fig. 2a-b) and turbulent flow (Fig. 2c, Supplementary Fig. 2c), the vascular lumen and the cardiac chambers were demonstrated with no signal intensity loss compared with the clinical sequence. The lumen of the pulmonary veins was sharply demarcated, reducing off-resonance artifacts (Fig. 2d-e), and could be traced in the lung parenchyma. Blurring from respiratory motion was attenuated with the proposed approach (Fig. 3a-d) with respect to the clinical sequence, resulting in improved vascular dimensioning and coronary depiction. Furthermore, non-rigid motion correction led to increased vessel sharpness and length in the delineation of the coronary arteries (3e). The research sequence generated robust 3DWH imaging in patients with high body mass index (BMI), high heart rate, prosthetic valves and devices and regurgitant lesions (Supplementary Fig. 1–2).

### 3.3 Qualitative image quality analysis

#### 3.3.1 Diagnostic accuracy and diagnostic confidence

Diagnostic confidence was higher for the iNAV-3DWH-PROST compared with the clinical sequence (mean±SD, 3.9 ± 0.2 vs 3.4 ± 0.8 p < 0.001) (Table 2). iNAV-3DWH-PROST achieved full segmental diagnoses with high or definite diagnostic confidence in all examinations but one case [98% (59/60) success rate], where the pulmonary veins and the coronary arteries could not be visualized due to artifact from coil for occlusion of arteriovenous collateral. The dNAV-3DWH clinical sequence failed to provide full sequential segmental analysis in seven cases [88% (53/60) success rate]. Failure was due to substantial artifact in pulmonary veins, pulmonary arteries in one case (flow and off-resonance artifacts), artifacts in the pulmonary and systemic veins, great arteries and coronary arteries, due to significant respiratory motion in two cases; artifacts in the left pulmonary veins in one case (off-resonance artifacts); substantial artifacts in the cardiac chambers, pulmonary veins and coronary arteries due to residual respiratory motion in two cases; significant artifacts from respiratory motion and coil in the pulmonary veins, systemic veins, coronary arteries and cardiac chambers in the last case. All seven diagnoses were confirmed with previous contrast-enhanced T2 prepared bSSFP and turbo-spin echo acquisitions in five participants, echocardiography in one participant and cardiac catheterization in the last participant.

**Table 2:** Comparison of the diagnostic confidence of the clinical dNAV-3DWH versus the research iNAV-3DWH-PROST.

	iNAV-3DWH-PROST	dNAV-3DWH	iNAV-3DWH-PROST vs dNAV-3DWH
Diagnostic confidence (All Reviewers)	3.9 ± 0.2	3.4 ± 0.8	P < 0.001
Reviewer 1	3.9 ± 0.3	3.4 ± 0.8	P = 0.005
Reviewer 2	4 ± 0.2	3.5 ± 0.8	P = 0.004
Reviewer 3	3.9 ± 0.2	3.6 ± 0.8	P = 0.03

Diagnostic sensitivity and specificity for the detection of pulmonary arteries abnormalities, coarctation, aortic aneurysm/dilatation, partial/total anomalous pulmonary venous drainage, coronary artery abnormalities and variations of the aortic arch and head and neck vessels was higher for the proposed iNAV-3DWH-PROST sequence, albeit with overlapping confidence intervals with the clinical sequence for all lesions, except for the diagnosis of variations in pulmonary venous return (Supplementary Table 3).

**Fig. 4:**
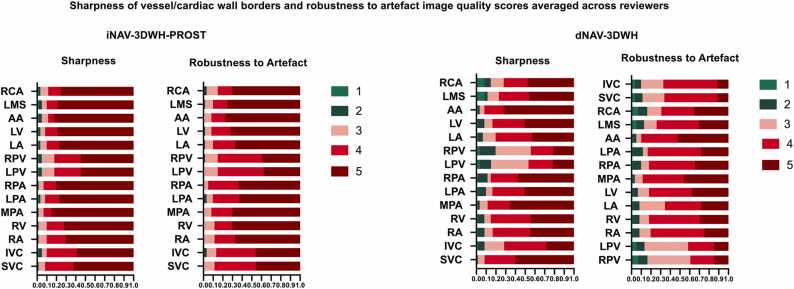
Bar plot demonstrating image quality scores for the research versus the clinical datasets. Bar plot demonstrating image quality scores for the research iNAV-3DWH-PROST versus the clinical dNAV-3DWH. Image quality scores with regards to sharpness of vessel/cardiac borders and robustness to artefact averaged across the three reviewers. Vessel sharpness and artefact scoring colour correspondence is provided next to the respective colour bar. The y-axis reflects percentage of datasets. A maximum image quality score of 5 for vessel sharpness indicates sharp delineation of all relevant anatomic structures with excellent contrast, whereas a robustness to artefact score of 5 reflects no ghosting, signal voids or cardiac motion blurring (Supplementary Table 2).

The iNAV-3DWH-PROST yielded superior image quality in terms of sharpness of endocardial and vascular wall and robustness to artefacts for the systemic and the pulmonary veins, the intracardiac structures, the pulmonary arteries and the coronary arteries. (Fig. 4), (Table 3).

**Table 3:** Image quality scores for wall sharpness and presence of artifact between the clinical and research datasets for the corresponding intrapericardial structures.

Structure	Sharpness of endocardial/vascular wall			Robustness to artifact		
	dNAV	iNAV	P value	dNAV	iNAV	P value
SVC	3.9 ± 1	4.6 ± 0.7	<0.001*	3.7 ± 0.9	4.4 ± 0.7	<0.001*
IVC	3.9 ± 1	4.5 ± 0.8	<0.001*	3.7 ± 0.9	4.3 ± 0.8	<0.001*
RA	4.2 ± 1	4.6 ± 0.8	0.004*	4.1 ± 0.9	4.6 ± 0.7	<0.001*
RV	4.2 ± 0.9	4.6 ± 0.8	0.006*	4.1 ± 0.9	4.6 ± 0.7	<0.001*
MPA	4.1 ± 0.9	4.7 ± 0.7	0.04*	4.4 ± 0.8	4.8 ± 0.6	0.005*
LPA	4.3 ± 1	4.7 ± 0.8	0.09	4 ± 1	4.5 ± 0.7	<0.001*
RPA	4.3 ± 1	4.7 ± 0.7	0.01*	4.1 ± 1	4.6 ± 0.6	<0.001*
LPV	3.4 ± 1	4.3 ± 0.9	<0.001*	3.3 ± 1	4.2 ± 0.7	<0.001
RPV	3.4 ± 1	4.3 ± 0.9	<0.001*	3.3 ± 1	4.2 ± 0.7	<0.001*
LA	4.1 ± 0.9	4.7 ± 0.8	<0.001*	3.9 ± 1	4.6 ± 0.7	<0.001*
LV	4.3 ± 0.9	4.7 ± 0.8	0.006*	4.2 ± 0.9	4.7 ± 0.7	<0.001*
AAo	4.6 ± 0.8	4.8 ± 0.7	0.3	4.5 ± 0.7	4.8 ± 0.6	0.08
RCA	4 ± 1.2	4.6 ± 0.8	<0.001*	3.9 ± 1.2	4.7 ± 0.8	<0.001*
LMS	4.1 ± 1.2	4.7 ± 0.8	<0.001*	4 ± 1.1	4.7 ± 0.7	<0.001*

Note: —Data presented as means ± standard deviations.

AAo= ascending aorta, IVC: inferior vena cava, LA: left Atrium, LPV: left pulmonary veins, LMS: left main stem, LV: left ventricle, MPA: main pulmonary artery, RA: right atrium, RCA = right coronary artery, RPV: right pulmonary veins, RV: right ventricle, SVC: superior vena cava

* P < 0.05, denoting statistical significance

### 3.4 Quantitative image quality analysis

The iNAV-3DWH-PROST had higher or comparable contrast ratio to the clinical native sequence for all the structures assessed (Supplementary Table 4).

### 3.5 Measurement reproducibility analysis

**Fig. 5:**
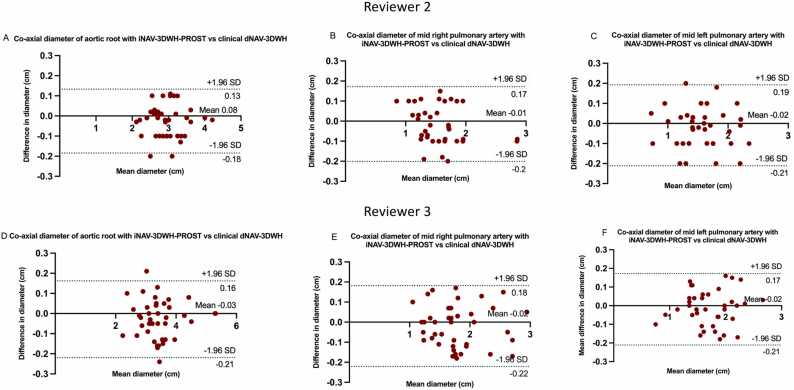
Bland-Altman plots for co-axial diameter measurements between the research and the clinical sequence for reviewer 2 and 3. Bland-Altman plots for co-axial diameter measurements of the aortic root, mid right and left pulmonary artery with the research iNAV-3DWH-PROST versus the clinical dNAV-3DWH for reviewer 2 (A, B, C) and 3 (D, E, F). The black line indicates the mean bias of the diameter measurements and the green line represents the 95% confidence interval. Values are given in cm. Measurements from both reviewers demonstrated good agreement between sequences for all three landmarks.

There was very good agreement in the co-axial diameter measurements at the defined landmarks between the proposed and the clinical sequence with a mean difference of less than 0.08 cm and narrow limits of agreement (<0.19 cm) for both reviewers (Fig. 5).

Intraclass correlation coefficients for intra- and interobserver agreement analysis for each rater is summarized in Supplementary Table 5. The mean difference between the inter- and intra-observer measurements at the defined landmarks with the proposed iNAV-3DWH-PROST sequence was less than 0.07 cm and narrow limits of agreement (<0.17 cm) (Supplementary Figure 2).

### 3.6 Qualitative analysis of the research framework pre- and post-PROST denoising

The low-rank patch-based based denoising contributed to significant attenuation of the artifacts in the cardiac chambers, the great arteries and the coronary arteries, when compared to the iNAV-3DWH dataset without PROST (Supplementary Table 6 and Supplementary Figure 4). The diagnostic performance was not altered by the addition of the denoising, and remained higher compared to the clinical dataset (Supplementary Table 3 & 7).

## 4 Discussion

Conventional MRI in congenital heart disease (CHD) is associated with long examination times and image quality degradation in view of respiratory motion, flow and off resonance artefacts.

In this work, we have proposed and validated a framework for accelerated and high-quality 3D whole-heart imaging in adult patients with CHD. It encompasses image-based navigation for translational and non-rigid respiratory motion correction, Cartesian undersampled acquisition in concert with low-rank based denoising and resilience to B0 and B1 field inhomogeneities with the application of additional 180° refocusing pulses in the MLEV4 T2-preparation scheme. We demonstrated that the proposed iNAV-3DWH-PROST can achieve 4- to 5-fold acceleration in the acquisition time (3.1 ± 0.9 min vs 13.9 ± 3.9 min, p < 0.001) and higher overall diagnostic confidence than the conventional sequence [3.9 ± 0.2 vs 3.4 ± 0.8, p < 0.001] along with reliable quantification of the vascular dimensions with excellent intra- and inter-reviewer agreement.

The proposed efficient iNAV-3DWH-PROST reduces scan, preparation time and complexity by being native and acquired under self-ventilatory, free-breathing status (no contrast material needed, no patient cannulation and no patient ventilation required). This has significant clinical implications, as CHD patients undergo repetitive MRI through their lifetime; hence, efficient and high-quality anatomical imaging can facilitate enhanced clinical decision-making, in addition to streamlining the clinical workflow and reduction of the healthcare cost. The significant attenuation of artefacts and enhanced contrast ratio is important for the generation of volume-rendered datasets and high-quality 3D models, as postprocessing for 3D virtual or printing relies primarily on thresholding based on signal intensity (Supplementary Fig 5) [27]. This could be an important clinical tool, that can aid pre-procedural understanding of complex anatomy. It can be useful for surgical decision making and simulation, computer-aided design of surgical baffles and patches, as well as hands-on surgical training and cardiovascular morphology teaching.

The significant improvement in image quality is attributed to the advanced motion correction and recontruction strategy in concert with the low-rank patch-based based denoising. Additional merit is potentially granted by the incorporation of four refocusing pulses in the T2 preparation scheme (MLEV4). Considering that MLEV4 is a simple adjustment to the clinical sequence, with no associated limitations to our knowledge and clinical experience, this was employed in the research sequence in this study.

Recent technical advances have resulted in additional promising techniques, that can achieve reliable diagnostic images in short acquisition times. Nguyen et al. [28] demonstrated in a multi-center study the feasibility of accelerated 3DWH and cine imaging in 12-min with a spatial resolution of 0.6–1.3 mm^3^ in paediatric patients with CHD; using the four-dimensional (4D) multiphase steady-state imaging with contrast enhancement (MUSIC) pulse sequence. However, the study necessitated the administration of ferumoxytol, which is not licensed in Europe. Moreover, the sequence has only been validated in a protocol that required general anesthesia with mechanical ventilatory support, which is very rarely performed in non-paediatric CHD. Mesropyan et al. [29], investigated the diagnostic utility of mDixon 3DWH in paediatric patients with CHD, employing a reconstruction framework which was based on a combination of compressed sensing and parallel imaging, with a mean acquisition time of 2 min. Nevertheless, this approach involved anisotropic acquisition, necessitated the administration of gadolinium-based contrast agent, sedation and prolonged preparation time, thus limiting widespread clinical adoption and patient comfort. Further advances introducing machine-learning in the reconstruction have been recently proposed, [30], [31] and bear the potential for significant acceleration in the clinical workflow. However, current applications are hardware demanding, requiring reconstruction on graphic processing units and further developments are anticipated for their integration in the clinical routine.

## 5 Limitations

Our study has limitations. This was a single-center study, including a moderate study sample size. The described motion-compensated research reconstruction was performed inline without GPU support, and denoising was done off-line. Updated versions of the described reconstruction featuring GPU support and an inline implementation of the denoising can be expected to perform significantly faster. Furthermore, the sequence type can be inferred from the images (coronal orientation for the iNAV-3DWH-PROST versus sagittal for the clinical); therefore, despite the anonymization and de-identification of the datasets, fully blinded evaluations were not feasible. However, the datasets were randomised between the readers to minimize bias. In our CHD tertiary center we do not often use gadolinium-based contrast agents in CHD patients and therefore there is no dataset acquired post contrast. Hence no comparison between the contrast-enhanced clinical sequence and the contrast-enhanced iNAV-3DWH-PROST was performed. This study cohort comprised adult patients and all the acquisitions were performed in mid-diastole. The performance of the framework in systolic acquisitions or dual-phase acquisition, where flow-artefacts in regurgitant and stenotic lesions might be more pronounced, needs to be evaluated in future work. Additionally, future experiments to investigate individually the contribution of MLEV4 T2-prep, dNAV and iNAV to the image quality and image contrast would be of interest. Finally, the integration of arrythmia detection and rejection algorithms, that could help prevent residual cardiac motion artifacts should be evaluated in future work.

## 6 Future work

Further assessment, through reproducibility studies between sites and vendors, including a larger patient group with paediatric patients, should be investigated in the future, to establish its clinical application for reliable and efficient anatomical imaging in CHD. In particular for pediatric patients, anticipated merits of the proposed framework include shorter scan time and hence shorter anesthetic time for neonates and younger children or use of sedation only, and improved compliance for adolescents. The attenuation of artefacts and the increased sharpness of vascular wall might also obviate the need for contrast-agent to vizualise smaller structures, e.g. coronary arteries and pulmonary veins. This will be investigated in future work.

## 7 Conclusion

The iNAV-3DWH-PROST framework achieved 3D whole-heart imaging in ∼3 min acquisition time and 3-min reconstruction time with robust image quality in adult patients with CHD. The proposed approach mitigated frequently encountered artefacts (off-resonance, flow-, respiratory-related). It achieved higher diagnostic confidence to the clinical sequence in four- to five-fold shorter scan time.

## Ethics approval and consent to participate

The study was approved by the local institutional review board at Guy’s and St Thomas; NHS Foundation Trust and the UK National Research Ethics Committee (Reference Number: 230350). No studies involving animals were performed. Written informed consent was obtained from all subjects according to institutional guidelines, and the study was approved by our institutional review board.

## Consent for publication

Written, informed consent was obtained from the patients for anonymised publication of their individual details and all Figures in this manuscript. The individual consent forms are held by the authors and the sponsoring institution and are available for review at the request of the JCMR editor-in-chief.

## Funding information

The authors acknowledge financial support from the BHF Centre of Research Excellence and 10.13039/501100000274BHF PG/18/59/33955 and RG/20/1/34802, 10.13039/501100000266EPSRC EP/P001009, EP/P032311/1, EP/P007619, Wellcome EPSRC Centre for Medical Engineering (NS/A000049/1), Fondecyt N° 1210638, Millennium Institute for Intelligent Healthcare Engineering ICN2021_004 and the Department of health via the National Institute for Health Research (NIHR) comprehensive Biomedical Research Centre award to Guy’s and St. Thomas’ NHS Foundation Trust. The views expressed are those of the authors and not necessarily those of the NHS, the NIHR, or the Department of Health.

## Author contributions

AF, CP and RMB were involved in the conception and design of this project. AF was involved with data acquisition, interpretation and post-processing, statistical and clinical analysis, and the generation of this manuscript. KP and CR were involved in the patient recruitment, the analysis and interpretation of data. CP, RMB, CV and CM were involved with MRI sequence development and image reconstruction. CP, RMB, KPK and RN were involved with the in-line implementation of the research sequence. All authors participated in the critical revision and final approval of this manuscript.

## CRediT authorship contribution statement

**Velasco Carlos:** Investigation, Methodology, Validation, Visualization, Writing – review & editing. **Neji Radhouene:** Methodology, Writing – review & editing. **Kunze Karl P:** Formal analysis, Writing – review & editing. **Botnar Rene M:** Conceptualization, Formal analysis, Investigation, Methodology, Writing – review & editing. **Prieto Claudia:** Conceptualization, Formal analysis, Investigation, Methodology, Writing – review & editing. **Fotaki Anastasia:** Conceptualization, Formal analysis, Supervision, Writing – original draft, Writing – review & editing. **Pushparajah Kuberan:** Formal analysis, Writing – review & editing. **Rush Christopher:** Formal analysis, Writing – review & editing. **Munoz Camila:** Investigation, Methodology, Validation, Visualization, Writing – review & editing.

## Declaration of Competing Interest

A.Fo: disclosed no relevant relationships, K.P.: disclosed no relevant relationships, CM: disclosed no relevant relationships, CR: disclosed no relevant relationships, CV: disclosed no relevant relationships, RN and KPK: are employees of SIEMENS Healthcare Limited, CV: disclosed no relevant relationships, RMB: disclosed no relevant relationships, CP: disclosed no relevant relationships. AF, KP, CR had control of the data collection and the derived information, so there was no conflict of interest for KPK and RN who are employees of SIEMENS Healthcare Limited.

## Data Availability

Data generated or analysed during the study are available from the corresponding author by request.
