# Supplementary material for: Highly efficient free-breathing 3D whole-heart imaging in 3-min: single center study in adults with congenital heart disease
Source: J Cardiovasc Magn Reson. 2023 Dec 22;26(1):100008. doi: 10.1016/j.jocmr.2023.100008 (PMC11211218; doi:10.1016/j.jocmr.2023.100008)
Supplement: Supplementary file 1 — Supplementary material. [file mmc1.docx]

**Supplementary Material**

**Supplementary Table 1**: Imaging parameters for the clinical dNAV-3DWH versus the research iNAV-3DWH-PROST

| **Sequence** | **dNAV-3DWH** | **iNAV-3DWH-PROST** |
| --- | --- | --- |
| Acceleration | GRAPPA 2 | VD-CASPR 4 |
| Resolution | 1.5mm^3^ | 1.5mm^3^ |
| Field of view | 400x256x134-202mm | 400x300x104-208 mm |
| T2 preparation | 40ms | 40ms |
| Respiratory motion | Diaphragmatic gating | Image-based navigation |
| Reconstruction | Inline (GRAPPA) | Inline (it-SENSE Motion Corrected) |
| Denoising | No | Offline-3D PROST |
| Number of slices, range | 89-134 | 70-138 |
| TR/TE, msec | 3.3/1.6 | 3.4/1.7 |
| Bandwidth, Hz/pixel | 557 | 919 |
| Flip angle ° | 90 | 90 |
| Fat Sat Flip Angle ° | 110 | 130 |
| Free-breathing | Yes | Yes |
| Contrast-enhanced | No | No |
| Orientation | Sagittal | Coronal |
| Diastolic window | 120-150msec (subject-specific) | 120-150msec (subject-specific) |

**Supplementary Table 2:** Data scoring criteria

| Diagnostic scores | 1 | 2 | 3 | 4 | 5 |
| --- | --- | --- | --- | --- | --- |
| Sharpness of vessel/cardiac wall borders | Non-diagnostic (insufficient visualisation) | Substantial limitations in wall definition | Moderate definition of the wall (diagnostic) | Good definition of the wall with mild limitation | Excellent definition of the wall |
| Robustness to artefact | Severe artefact (non-diagnostic) | Significant artefact degrading image quality (non-diagnostic) | Moderate artefact (diagnostic) | Mild artefact not interfering with diagnosis | Minimal artefact |

**Supplementary Table 3:** Comparison of the diagnostic sensitivity, specificity and accuracy between the research iNAV-3DWH-PROST and the clinical dNAV-3DWH datasets

|  | Sensitivity | | Specificity | | Accuracy | | |
| --- | --- | --- | --- | --- | --- | --- | --- |
|  | iNAV-  3DWH-PROST | dNAV-3DWH | iNAV-  3DWH-PROST | dNAV-3DWH | iNAV-  3DWH-PROST | dNAV-3DWH |  |
| MPA stenosis | 100 (1/1) [17, 100] | 100 (1/1) [1, 100] | 100 (59/59) [93, 100] | 97 (57/59) [88,100] | 100 (60/60) [93,100] | 95 (58/60) [86,99] |  |
| RPA stenosis | 100 (6/6) [56, 100] | 67 (4/6) [22,96] | 100 (54/54) [92, 100] | 100 (54/54) [92, 100] | 100 (60/60) [93,100] | 97 (58/60) [88,100] |  |
| LPA stenosis | 100 (6/6) [56, 100] | 67 (4/6) [22,96] | 100 (54/54) [92, 100] | 100 (54/54) [92, 100] | 100 (60/60) [93,100] | 97 (58/60) [88,100] |  |
| Coarctation | 100 (3/3) [29,100] | 100 (3/3) [29,100] | 100 (57/57) {92, 100] | 96 (55/57) [87, 100] | 100 (60/60) [93,100] | 97 (58/60) [88,100] |  |
| Aortic aneurysm/  dilatation | 100 (11/11) [70, 100] | 100 (11/11) [70, 100] | 100 (49/49) [91, 100] | 96 (47/49) [86, 99] | 100 (60/60) [93,100] | 97 (58/60) [88,100] |  |
| Anomalous pulmonary venous drainage | 100 (2/2) [29-100] | 0 (0/2) [0, 71] | 97 (56/58) [88, 100] | 78 (45/58) [65, 86] | 97 (58/60) [88,100] | 75 (45/60) [63,84] |  |
| Coronary abnormalities | 60 (3/5) [23,88] | 7 (1/5) [2, 64] | 100 (55/55) [92, 100] | 80 (44/55) [67, 89] | 97 (58/60) [88,100] | 75 (45/60) [63,84] |  |
| Variations of aortic arch and head and neck vessels | 100 (19/19) [80,100] | 95 (18/19) [74, 100] | 100 (41/41) [90, 100] | 98 (40/41) [86, 100] | 100 (60/60) [93,100] | 97 (58/60) [88,100] |  |

Note: % (raw data) [95% confidence interval]

**Supplementary Table 4:** Comparison of the contrast ratio between the clinical dNAV-3DWH and the iNAV-3DWH-PROST for the intrapericardiac structures

| Structure | Contrast Ratio | | |
| --- | --- | --- | --- |
|  | dNAV | iNAV | P value |
| SVC | 0.5±0.2 | 0.9±0.5 | <0.001* |
| IVC | 0.5±0.5 | 1.0±0.5 | <0.001* |
| RA | 0.5±0.2 | 1.2±0.5 | <0.001* |
| RV | 1.5±0.7 | 1.4.±0.4 | 0.2 |
| MPA | 1.5±0.6 | 1.6.±0.6 | 0.8 |
| LPV | 0.5±0.6 | 0.6±0.4 | 0.2 |
| RPV | 0.3±0.4 | 0.5±0.4 | 0.09 |
| LA | 1.1±0.7 | 0.9.±0.4 | 0.06 |
| LV | 1.4±0.9 | 1.3±0.4 | 0.4 |
| AA | 1.8±0.8 | 1.7±0.5 | 0.3 |

Note.—Data presented as means ± standard deviation.

AA= ascending aorta, IVC: inferior vena cava, LA: left Atrium, LPV: left pulmonary veins, LV: left ventricle, MPA: main pulmonary artery, RA: right atrium, RPV: right pulmonary veins, RV: right ventricle, SVC: superior vena cava.

* P<0.05, denoting statistical significance

**Supplementary Table 5:** Intra-Class-Correlation inter-reviewer and intra-reviewer agreement values and 95% Confidence Intervals

| Structure | Inter-observer variability | Intra-observer variability |
| --- | --- | --- |
| Aortic root | 0.98 (0.97, 0.99) | 0.99 (0.99, 1) |
| Right pulmonary artery | 0.99 (0.98, 1) | 0.99 (0.98, 1) |
| Left pulmonary artery | 0.99 (0.97, 0.99) | 0.99 (0.98, 1) |

Note.—Data presented as average measure ICC with 95% CI in parentheses.

CI: Confidence Interval, ICC: Intraclass Correlation Coefficient

**Supplementary Table 6:** Image quality scores for wall sharpness and presence of artifact between the clinical and research datasets for the corresponding intrapericardial structures

| Structure | Sharpness of vascular/endocardial borders | | | | | Robustness to artefact | | | | |
| --- | --- | --- | --- | --- | --- | --- | --- | --- | --- | --- |
|  | dNAV-3DWH | iNAV-3DWH | iNAV-3DWH-PROST | iNAV-3DWH-PROST vs  iNAV-3DWH  p value | iNAV-3DWH vs dNAV-3DWH  p value | dNAV-3DW | iNAV-3DWH | iNAV-3DWH-PROST | iNAV-3DWH-PROST vs  iNAV-3DWH  p value | iNAV-3DWH vs dNAV-3DWH  p value |
| SVC | 3.9±1 | 4.5±0.9 | 4.6±0.7 | 0.9 | 0.001* | 3.7±0.9 | 4.3±0.8 | 4.4±0.7 | 0.7 | 0.004* |
| IVC | 3.9±1 | 4.5±0.9 | 4.5±0.8 | 0.9 | 0.001* | 3.7±0.9 | 4.3±0.8 | 4.3±0.8 | 0.8 | 0.02* |
| RA | 4.2±1 | 4.5±0.9 | 4.6±0.8 | 0.8 | 0.1 | 4.1±0.9 | 4.3±0.8 | 4.6±0.7 | 0.03* | 0.5 |
| RV | 4.2±0.9 | 4.5±0.8 | 4.6±0.8 | 0.9 | 0.1 | 4.1±0.9 | 4.3±0.7 | 4.6±0.7 | 0.01* | 0.7 |
| MPA | 4.1±0.9 | 4.5±0.9 | 4.7±0.7 | 0.7 | 0.02* | 4.4±0.8 | 4.3±0.8 | 4.8±0.6 | 0.001* | 0.8 |
| LPA | 4.3±1 | 4.6±0.8 | 4.7±0.8 | 0.6 | 0.2 | 4±1 | 4.1±0.8 | 4.5±0.7 | 0.001* | 0.6 |
| RPA | 4.3±1 | 4.6±0.8 | 4.7±0.7 | 0.9 | 0.1 | 4.1±1 | 4.2±0.8 | 4.6±0.6 | 0.004* | 0.6 |
| LPV | 3.4±1 | 4.3±0.9 | 4.3±0.9 | 0.8 | 0.001* | 3.3±1 | 3.9±0.8 | 4.2±0.7 | 0.8 | 0.001* |
| RPV | 3.4±1 | 4.3±0.9 | 4.3±0.9 | 0.8 | 0.001* | 3.3±1 | 3.9±0.8 | 4.2±0.7 | 0.6 | 0.001* |
| LA | 4.1±0.9 | 4.6±0.8 | 4.7±0.8 | 0.7 | 0.02* | 3.9±1 | 4.2±0.7 | 4.6±0.7 | 0.004* | 0.4 |
| LV | 4.3±0.9 | 4.6±0.8 | 4.7±0.8 | 0.6 | 0.1 | 4.2±0.9 | 4.3±0.8 | 4.7±0.7 | 0.001* | 0.9 |
| AAo | 4.6±0.8 | 4.6±0.9 | 4.8±0.7 | 0.3 | 0.9 | 4.5±0.7 | 4.4±0.8 | 4.8±0.6 | 0.001* | 0.3 |
| RCA | 4±1.2 | 4.4±1 | 4.6±0.8 | 0.5 | 0.05* | 3.9±1.2 | 4.2±0.9 | 4.7±0.8 | 0.001* | 0.09 |
| LMS | 4.1±1.2 | 4.5±1 | 4.7±0.8 | 0.7 | 0.02* | 4±1.1 | 4.3±0.8 | 4.7±0.7 | 0.04* | 0.2 |

**Supplementary Table 7:** Comparison of the diagnostic sensitivity, specificity and accuracy between the research iNAV-3DWH-PROST and the iNAV-3DWH datasets

|  | Sensitivity | | Specificity | | Accuracy | | |
| --- | --- | --- | --- | --- | --- | --- | --- |
|  | iNAV-  3DWH-PROST | iNAV-3DWH | iNAV-  3DWH-PROST | iNAV-3DWH | iNAV-  3DWH-PROST | iNAV-3DWH |  |
| MPA stenosis | 100 (1/1) [17, 100] | 100 (1/1) [17, 100] | 100 (59/59) [93, 100] | 100 (59/59) [93, 100] | 100 (60/60) [93,100] | 100 (60/60) [93,100] |  |
| RPA stenosis | 100 (6/6) [56, 100] | 100 (6/6) [56, 100] | 100 (54/54) [92, 100] | 100 (54/54) [92, 100] | 100 (60/60) [93,100] | 100 (60/60) [93,100] |  |
| LPA stenosis | 100 (6/6) [56, 100] | 100 (6/6) [56, 100] | 100 (54/54) [92, 100] | 100 (54/54) [92, 100] | 100 (60/60) [93,100] | 100 (60/60) [93,100] |  |
| Coarctation | 100 (3/3) [29,100] | 100 (3/3) [29,100] | 100 (57/57) {92, 100] | 100 (57/57) {92, 100] | 100 (60/60) [93,100] | 100 (60/60) [93,100] |  |
| Aortic aneurysm/  dilatation | 100 (11/11) [70, 100] | 100 (11/11) [70, 100] | 100 (49/49) [91, 100] | 100 (49/49) [91, 100] | 100 (60/60) [93,100] | 100 (60/60) [93,100] |  |
| Anomalous pulmonary venous drainage | 100 (2/2) [29-100] | 100 (2/2) [29-100] | 97 (56/58) [88, 100] | 97 (56/58) [88, 100] | 97 (58/60) [88,100] | 97 (58/60) [88,100] |  |
| Coronary abnormalities | 60 (3/5) [23,88] | 60 (3/5) [23,88] | 100 (55/55) [92, 100] | 100 (55/55) [92, 100] | 97 (58/60) [88,100] | 97 (58/60) [88,100] |  |
| Variations of aortic arch and head and neck vessels | 100 (19/19) [80,100] | 100 (19/19) [80,100] | 100 (41/41) [90, 100] | 100 (41/41) [90, 100] | 100 (60/60) [93,100] | 100 (60/60) [93,100] |  |

**Supplementary figure 1 title:** Visual comparison of images from the research and clinical datasets from selected patients.

**Supplementary figure 1 legend:** Comparison of research iNAV-3DWH-PROST and clinical dNAV-3DWH has demonstrated significant scan time reduction and robustness in different underlying pathologies: **a:** Multiplanar reformatted images in 39-year-old man with transposition of the great arteries and subpulmonary LVOT and common origin of the RCA and LCx, post Senning procedure. Increased BMI (27) did not affect image quality of the iNAV-3DWH-PROST dataset, which had significant reduction of flow artefacts in the systemic venous baffles in comparison to the clinical sequence, **b:** Multiplanar reformatted images in a 39-year old man diagnosed with pulmonary stenosis (heart rate: 95bpm), **c:** Multiplanar reformatted images in a 39-year-old man with absent left atrioventricular connection and pulmonary atresia post fenestrated TCPC completion and device fenestration closure. The device does not cause significant artefacts in the iNAV-3DWH-PROST dataset, **d:** Multiplanar reformatted images in a 26-year-old woman with pulmonary stenosis post repair with bioprosthetic valve in the pulmonary position, **e:** Multi-planar reformatted images in a 20-year-old woman with dysplastic mitral valve post repair with mitral ring. The foreign material does not induce significant artefacts in the iNAV-3DWH-PROST dataset (pink arrow) in comparison to the clinical dataset (blank pink arrow).

Footnote: LCx: left circumflex, RCA: right coronary artery

**Supplementary figure 2 title:** Visual comparison of images from the research and clinical datasets from selected patients with regurgitant lesions.

**Supplementary figure 2 legend:** Comparison of research iNAV-3DWH-PROST and clinical dNAV-3DWH improved image quality in regurgitant lesions along with significant scan time reduction: **a:** Multiplanar reformatted images in 63-year-old man with perimembranous ventricular septal defect and concomitant aortic regurgitation. Signal loss artefact can be observed in both sequences, and can be useful to assess the defect (red arrow), albeit significant less artefact in the blood pool/myocardium interface (red arrowhead) is observed in the proposed approach **b:** Multiplanar reformatted images in a 19-year old woman diagnosed with tetralogy of Fallot and concomitant aortic regurgitation. The regurgitant jet in the clinical sequence creates increased signal loss in the blood pool compared to the proposed approach (red arrow). The left main stem is visualised with less blurring (red arrowhead) and motion artefacts are attenuated (yellow arrows) in the research sequence , **c:** Multiplanar reformatted images in a 25-year-old man with coarctation of the aorta and severe aortic stenosis, post Ross procedure. Signal void in the right ventricular outflow tract and main pulmonary artery due to current severe regurgitation in the pulmonary homograft (red arrows). Signal voids are accentuated in the conventional approach.

**Supplementary Figure 3 title:** Bland-Altman plots for co-axial diameter measurements with the research sequence for inter-reviewer agreement for reviewer 2 and 3.

**Supplementary Figure 3 legend:** Bland-Altman plots for co-axial diameter measurements of the aortic root, mid right and left pulmonary artery with the research iNAV-3DWH-PROST for inter-reviewer agreement between reviewer 2 and 3 (A, B, C) and for intra-reviewer agreement for reviewer 3 (D, E, F). The black line indicates the mean bias of the diameter measurements, and the green line represents the 95% confidence interval. Values are given in cm. Measurements demonstrated good intra-reviewer and intra-reviewer agreement for the iNAV-3DWH-PROST for all three landmarks.

**Supplementary Figure 4 title:** Visual comparison of images from the research datasets pre- (iNAV-3DWH) and post- PROST denoising (iNAV-3DWH-PROST)

**Supplementary Figure 4 legend: a:** Comparison of research datasets post-PROST denoising versus pre-PROST denoising **a:** Multiplanar reformatted images in 41-year-old man with congenitally-corrected transposition of the great arteries. The left anterior descending coronary artery is demarcated with less artefact post-PROST denoising (red arrows). **b:** Multiplanar reformatted images in a 47-year old woman diagnosed with total anomalous pulmonary venous drainage. The intracardiac anatomy is visualised with very similar quality in both research datasets. **c:** Multiplanar reformatted images in a 39-year-old man with transposition of the great arteries and left ventricular outflow tract obstruction post Senning procedure. Artefacts from turbulent flow in the main and branch pulmonary arteries (red arrowheads) are attenuated post-PROST denoising.

**Supplementary Figure 5 title:** 3D volume-rendered dataset acquired with the research and the clinical sequence

**Supplementary Figure 5 legend: a:** 3D volume-rendered dataset with the iNAV-3DWH-PROST versus the clinical dNAV-3DWH demonstrates improved delineation of the right coronary artery (arrowhead) and the systemic veins (red and white arrows) in a 19-year old with hypoplastic left heart syndrome post Fontan palliation, **b:** 3D volume-rendered dataset with the iNAV-3DWH-PROST versus the clinical dNAV-3DWH demonstrates improved delineation of the right and left coronary arteries (arrowheads), the systemic veins (red arrowheads) and the right atrium (white arrow) in a 39-year old man diagnosed with transposition of the great arteries and common origin of the coronary arteries.

Footnote: LCx: left circumflex, RCA: right coronary artery
