# Supplementary figures and images for: Highly efficient free-breathing 3D whole-heart imaging in 3-min: single center study in adults with congenital heart disease
Source: J Cardiovasc Magn Reson. 2023 Dec 22;26(1):100008. doi: 10.1016/j.jocmr.2023.100008 (PMC11211218; doi:10.1016/j.jocmr.2023.100008)

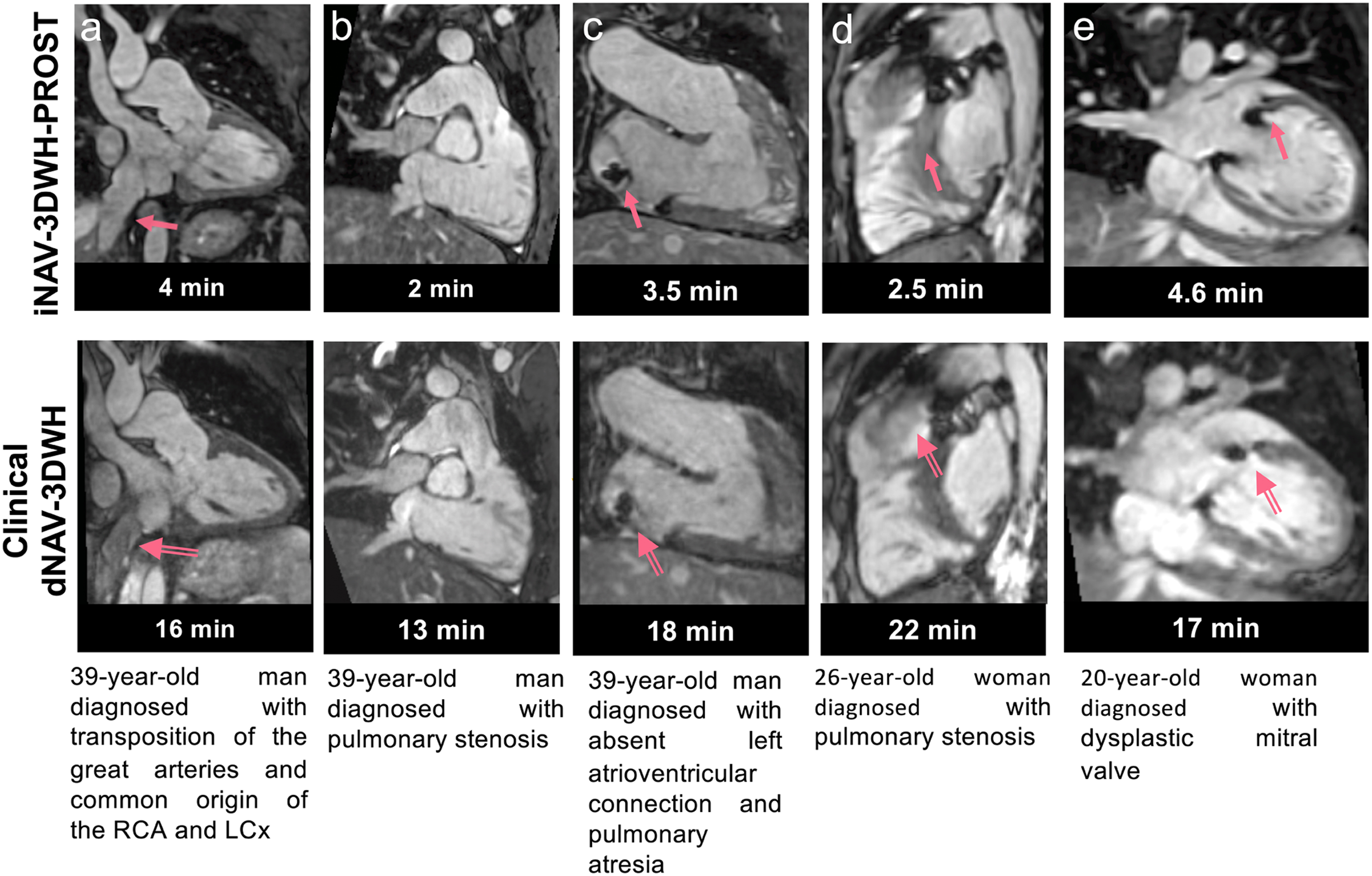

Supplement: Supplementary file 2 — Supplementary material. [file mmc2.jpg]

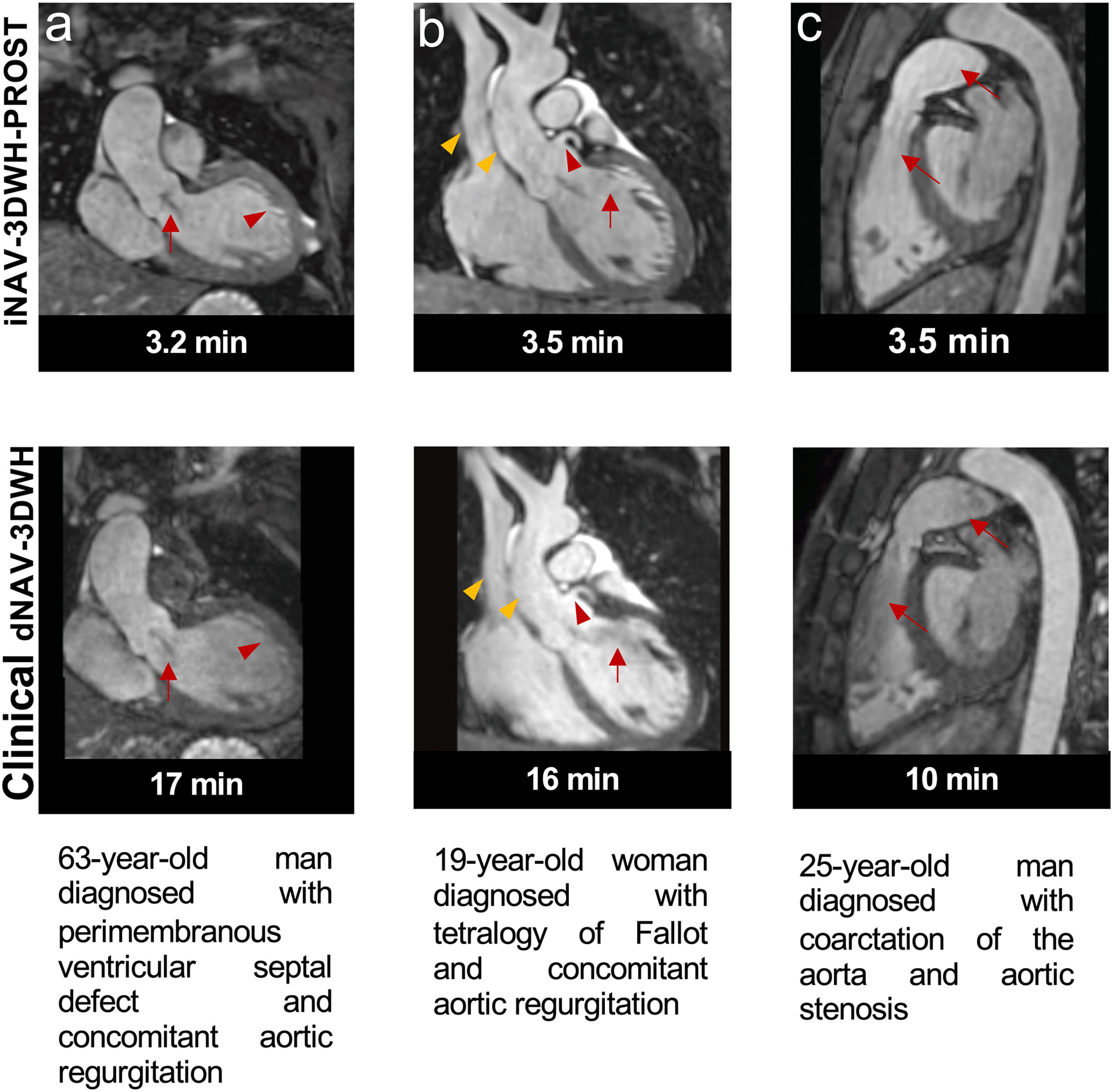

Supplement: Supplementary file 3 — Supplementary material. [file mmc3.jpg]

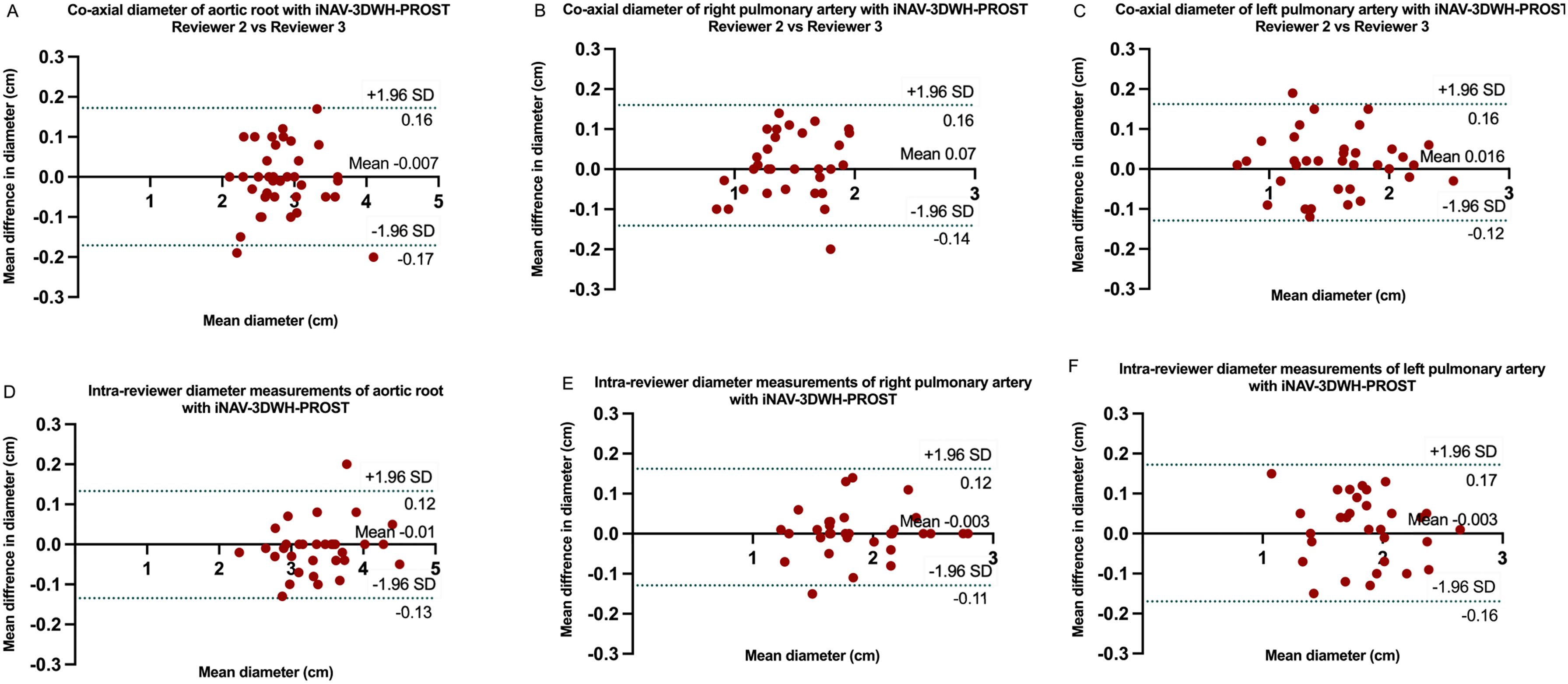

Supplement: Supplementary file 4 — Supplementary material. [file mmc4.jpg]

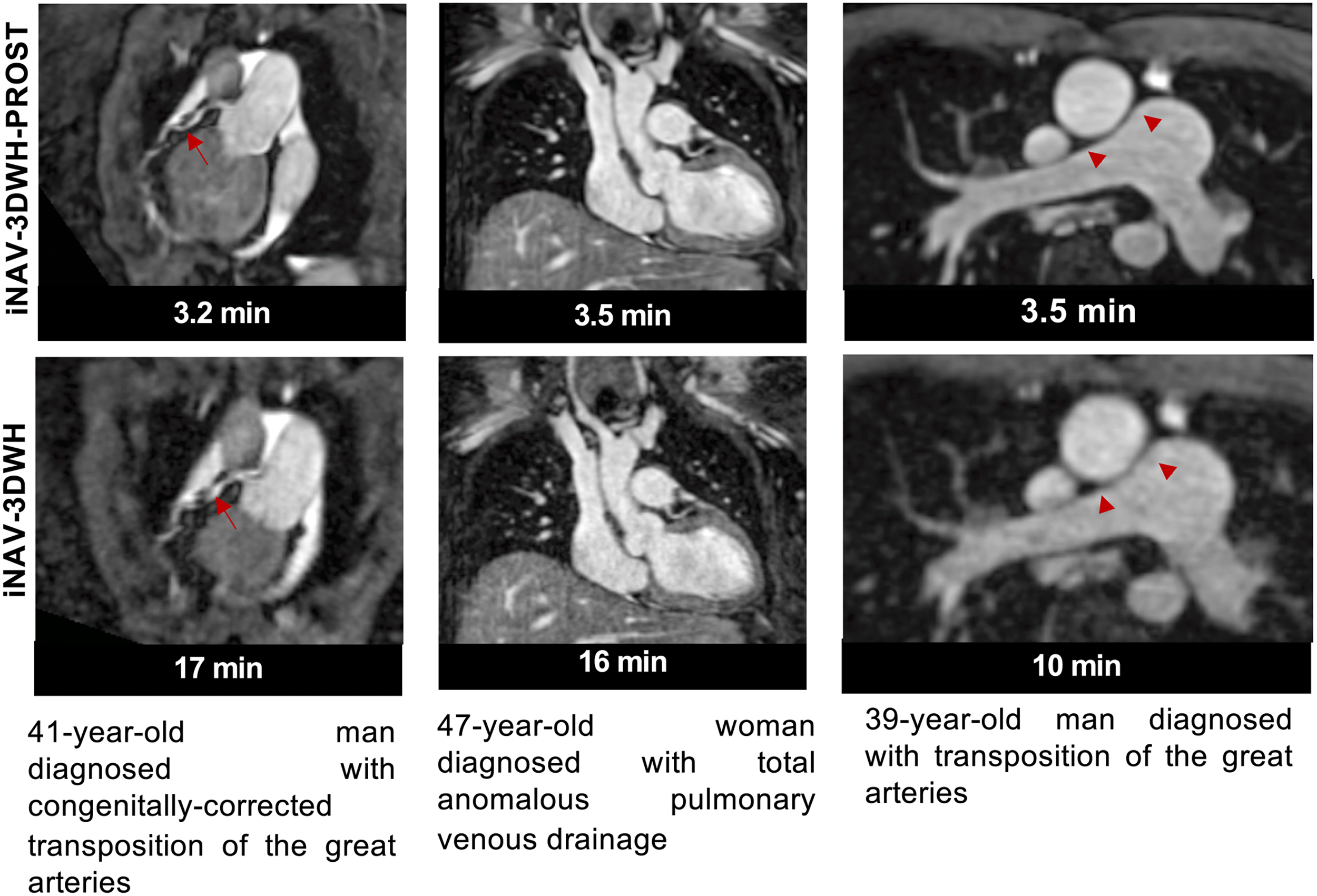

Supplement: Supplementary file 5 — Supplementary material. [file mmc5.jpg]

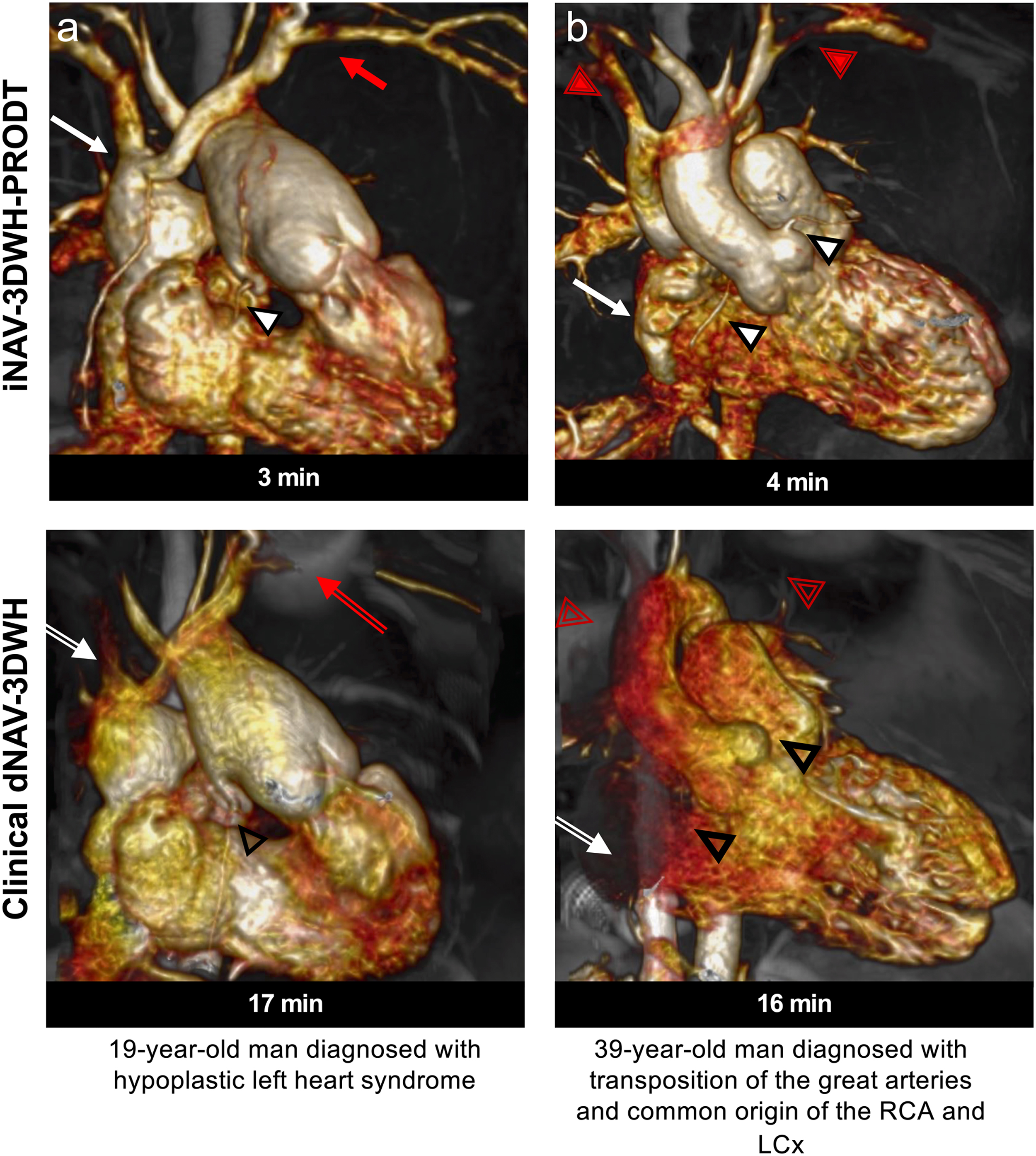

Supplement: Supplementary file 6 — Supplementary material. [file mmc6.jpg]
